# The MYTHEN detector for X-ray powder diffraction experiments at the Swiss Light Source

**DOI:** 10.1107/S0909049510026051

**Published:** 2010-07-22

**Authors:** Anna Bergamaschi, Antonio Cervellino, Roberto Dinapoli, Fabia Gozzo, Beat Henrich, Ian Johnson, Philipp Kraft, Aldo Mozzanica, Bernd Schmitt, Xintian Shi

**Affiliations:** a Paul Scherrer Institut, CH-5232 Villigen, Switzerland

**Keywords:** detectors, powder diffraction

## Abstract

The MYTHEN single-photon-counting silicon microstrip detector has been developed at the Swiss Light Source for time-resolved powder diffraction experiments. An upgraded version of the detector has been installed at the SLS powder diffraction station allowing the acquisition of diffraction patterns over 120° in 2θ in fractions of seconds. Thanks to the outstanding performance of the detector and to the calibration procedures developed, the quality of the data obtained is now comparable with that of traditional high-resolution point detectors in terms of FWHM resolution and peak profile shape, with the additional advantage of fast and simultaneous acquisition of the full diffraction pattern. MYTHEN is therefore optimal for time-resolved or dose-critical measurements. The characteristics of the MYTHEN detector together with the calibration procedures implemented for the optimization of the data are described in detail. The refinements of two known standard powders are discussed together with a remarkable application of MYTHEN to organic compounds in relation to the problem of radiation damage.

## Abstract 2

A report on the characterization, calibration and performances of the MYTHEN photon-counting silicon microstrip detector at the powder diffraction station at the Swiss Light Source is given.

## 1. Introduction

X-ray powder diffraction (XRPD) allows rapid non-destructive analysis of multi-component mixtures and of materials not available in single crystals and the study of industrial compounds in the same microcrystalline form as the final product (Tremayne, 2004 ▶). Furthermore, non-ambient XRPD analysis is often more successful than using single crystals owing to the difficulties in preserving the quality of a single crystal during the phase transformations.

Since a powder is formed by a very large number of microcrystals, ideally all possible crystal orientations are measured simultaneously and the three-dimensional reciprocal lattice is projected onto a one-dimensional space. The reduction of the entire reciprocal lattice into one dimension limits the data volume, simplifies the data collection strategy, and reduces the overall measurement time even for small and weakly scattering samples, opening opportunities for time-resolved studies.

However, these advantages are often at the expense of the ease of analysis and interpretation of the data. Crystal structure determination is especially complicated by the overlap of reflections in a powder diffraction pattern. For this reason XRPD has been traditionally used only to analyze the phase composition of samples of known crystal structure (fingerprinting) or to follow the dependence of the cell parameters on external conditions (*e.g.* temperature, pressure).

Structural solution with powder data has, however, greatly improved in the last 10 to 15 years. Developments in instrumentation, computer technology and powder diffraction experimental techniques [*e.g.* anisotropic thermal expansion and texture methods by Brunelli *et al.* (2003 ▶) and Wessels *et al.* (1999 ▶)] and methodologies [*e.g.* global optimization techniques by David & Shankland (2008 ▶); resolution bias algorithm by Altomare *et al.* (2009 ▶); charge flipping by Oszlányi *et al.* (2006 ▶)] implemented to strengthen the power of direct methods have all contributed to this success.

The advent of synchrotron sources has caused powder diffraction methods to enter a new era of development (Sakata *et al.*, 2007 ▶). The collimation and monochromaticity of the X-ray beam allow for an improvement in the angular resolution of the acquired patterns compared with conventional laboratory sources, whereas the high brilliance of the sources reduces measurement times by several orders of magnitude, allowing the study of the dynamics of samples on the time scale of fractions of a second.

### 1.1. Synchrotron radiation detectors for powder diffraction

The improvements in the radiation source must be accompanied by improved performances of radiation detectors. In order to fully exploit the advantages of synchrotron radiation powder diffraction experiments, stringent specifications are required for the X-ray detectors, which can be summarized as follows: *dynamic range* larger than 10^5^; *intrinsic angular resolution* better than 0.01°; *time resolution* better than 0.1 s on angular ranges larger than 40°.

Although CCDs (Svensson *et al.*, 1997 ▶; Mezouar *et al.*, 2005 ▶), image plates (Sarin *et al.*, 2009 ▶) or a-Si flat panels (Lee, Aydiner *et al.*, 2008 ▶) are often successfully employed, their limited dynamic range does not allow the correct observation of the intensity ratios between the strong and the weak reflections characterizing a powder pattern. In the following only photon-counting systems, which satisfy the requirements regarding the dynamic range (Lewis, 2003 ▶), will be discussed.

Powder diffraction beamlines are normally equipped with scintillator-based crystal analyzer detectors, which represent the state of the art in terms of angular (FWHM) resolution (Fitch, 2004 ▶; Gozzo *et al.*, 2006 ▶). They can reach resolutions of the order of 1 mdeg in 2θ, depending on the beam energy resolution and degree of collimation of the photon beam. However, their performance can only be improved at the expense of counting efficiency. Furthermore, point detectors record the diffraction patterns by scanning 2θ, which makes them intrinsically incompatible with time-resolved XRPD. Multichannel systems considerably reduce the acquisition time from hours to minutes (Hodeau *et al.*, 1998 ▶; Lee, Shu *et al.*, 2008 ▶; Tartoni *et al.*, 2008 ▶), but they still remain slow for most of the time-resolved measurements and they do not allow the monitoring of any radiation damage that may take place while the detector is being scanned (see §4.2).

In order to limit the duration of the measurements, detectors covering a large angular range and acquiring data in parallel over several electronics channels have been developed. Gas-based detector systems covering up to 60° allow frame rates of several hundreds of frames per second. However, the limited granularity of such detectors reduces the angular resolution to worse than 0.1° in the case of purely counting systems (Bateman *et al.*, 2007 ▶, 2008 ▶) or down to 0.06° in the case of an interpolating analog readout (Berry *et al.*, 2003 ▶).

The only detectors presently able to offer the required angular resolution over a large range are segmented semiconductor detectors. Two-dimensional detectors are sometimes used for textured samples or stress–strain measurement (Basolo *et al.*, 2007 ▶), but the existing systems are either limited in pixel size (Kraft *et al.*, 2009 ▶) or in active area (de Vries *et al.*, 2007 ▶).

The MYTHEN (Microstrip sYstem for Time-rEsolved experimeNts) detector has been developed at the Swiss Light Source (SLS) and is based on a one-dimensional microstrip silicon detector (Schmitt *et al.*, 2003 ▶, 2004 ▶).

A first version of the system covering an angular range of 60° in 2θ began operation for the powder diffraction users at the SLS in 2001, opening new perspectives for *in situ* studies with acquisition times of a few seconds (Budrovic *et al.*, 2004 ▶; Weyer *et al.*, 2005 ▶; Rosciano *et al.*, 2007 ▶). However, the front-end electronics still presented some limitations which strongly restricted the maximum count rate and the minimum detectable photon energy. Therefore a new version of the detector was developed.

The MYTHEN detector system has been replaced with an upgraded version which now covers 120° and has been in operation since summer 2007 (Bergamaschi *et al.*, 2009 ▶). Large MYTHEN detector systems and single modules have also been delivered by the Paul Scherrer Institut (PSI) to other synchrotron facilities (Marchal *et al.*, 2009 ▶; Haverkamp & Wallwork, 2009 ▶). The detector is now marketed by the DECTRIS spin-off company (http://www.dectris.com/).

## 2. Instruments and methods

### 2.1. The SLS powder diffraction station

The SLS is a third-generation synchrotron facility (2.4 GeV, 400 mA) at the Paul Scherrer Institute. The Material Science (MS) beamline has been operating since the opening of the SLS and it hosts two main experimental stations: powder diffraction and *in situ* surface diffraction (Gozzo et al., 2004 ▶; Patterson, Abela *et al.*, 2005 ▶; Patterson, Brönnimann *et al.*, 2005 ▶).

The wiggler source of the MS beamline produces a continuous spectrum of photons in the range 5–40 keV with a maximum flux of about 10^13^ photons s^−1^ at 12 keV and an angular acceptance of 0.23 mrad vertical by 2.5 mrad horizontal.

The beamline optics consists of a first mirror which provides vertical collimation and removes high-order harmonics, a double-crystal monochromator with an inherent energy resolution of 0.014%, which can also focus the beam horizontally using a sagitally focusable second crystal, and a second mirror with variable curvature which can either deliver a collimated beam for ultra-high-resolution XRPD or a vertically focused beam at the experimental station in use. The focused spot size at the XRPD station can be pushed down to 160 µm vertical by 450 µm horizontal.

The powder diffraction station is located in the first experimental hutch. The diffraction plane is vertical, and the sample and detector rotation stages have an absolute angular accuracy of ±1 arcsec.

Two 2θ stages carry independent detector systems. For high-resolution studies (≳2 mdeg FWHM in 2θ), a fivefold Si(111) crystal-analyzer/scintillator detector allows parallel detection with a nominal 2° 2θ separation between adjacent channels and a maximum count rate of the order of 1 MHz.

The second detector system is MYTHEN which is based on a silicon microstrip sensor with an Application-Specific Integrated Circuit (ASIC) operating in single-photon-counting mode.

**Figure 1:**
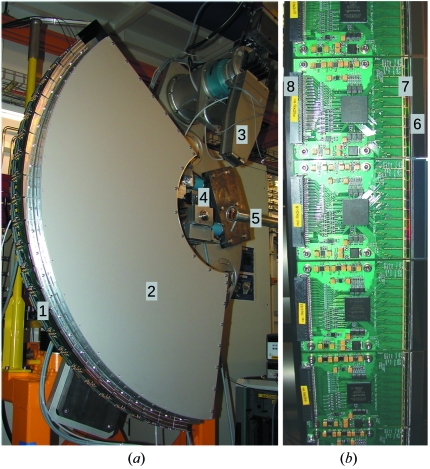
(*a*) Photograph of the MYTHEN detector installed at the powder diffraction station at the SLS and (*b*) a zoom on the modules building the detector. The numbers indicate the main elements of interest: (1) MYTHEN detector layer; (2) He-filled box behind which is fixed the data acquisition system; (3) analyzer crystal detector; (4) center of the diffractometer; (5) beampipe; (6) silicon microstrip sensor; (7) front-end electronics; (8) connector to the data acquisition system.

Fig. 1(*a*) ▶ shows the detection system installed at the SLS, covering 120° in 2θ.

The MYTHEN design is modular so that systems of different angular ranges can be assembled, as shown in Fig. 1(*b*) ▶. The modules are positioned at a distance of 76 cm from the sample and a He-filled box in between reduces the air absorption and scattering of the diffracted X-rays. Each module consists of 1280 independent channels and covers an angular range of 4.83° (*i.e.* about 265 strips per degree), with a gap of 0.17° (42.5 channels) between two neighboring modules. Since the detector is mounted on one of the diffractometer arms, it is possible to move it in order to acquire patterns in different positions and merge the data sets to avoid the data loss in the gaps between the modules.

Furthermore, powder diffraction patterns can be acquired up to 155° in 2θ which at 40 keV allows measurements up to *Q*-values of approximately 40 Å^−1^ making MYTHEN ideal for pair distribution function experiments (Cerny *et al.*, 2009 ▶).

### 2.2. The MYTHEN module

MYTHEN is based on microstrip sensors (Lutz, 1999 ▶) consisting of depleted high-resistivity 300 µm-thick n-doped silicon wafers segmented on one side by optical lithography into 1280 50 µm-pitch 8 mm-long p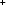-doped strips, each behaving like a reverse-biased diode. The backplane of the sensor consists of a thin aluminized surface and of a n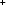-doped layer (∼2 µm) to provide a good electric contact for biasing the sensor and collecting the electrons.

The X-ray radiation is absorbed in the silicon mainly by the photoelectric effect and creates electron–hole pairs in the silicon bulk which then drift to the collection electrodes (the holes to the strips and the electrons to the backplane) under the influence of a strong electric field. Each X-ray photon of energy *E*_0_ produces a charge *Q* which is of the order of a few thousands of electrons (*Q* = *E*_0_/3.6 eV for Si) and is sufficient for the front-end electronics to count each photon directly (Mikulec, 2003 ▶).

The sensor is back illuminated, *i.e.* the radiation comes from the side opposite to the strip implants, to provide a uniform absorption efficiency. The charge produced by the X-rays converting in the backplane layers recombines before drifting to the strips owing to the absence of electric field in this region, reducing the detection efficiency in particular for the low X-ray energies. For the X-rays absorbed in the depleted n-doped silicon bulk, the holes left in the valence band of the silicon crystal drift with little diffusion toward the closest strip, so that the spatial resolution is essentially defined by the 50 µm strip pitch.

The efficiency of the sensor is more than 85% for X-ray energies in the range 5–10 keV and drops to about 25% at 20 keV, limited by the thickness of the silicon wafer.

The readout is carried out by a 128-channel ASIC directly wirebonded to the sensor (Mozzanica *et al.*, 2009 ▶). The ASIC is designed in radiation hard 0.25 µm UMC technology and is expected to support an integrated dose up to tens of Mrad (Sobott *et al.*, 2009 ▶) like the PILATUS II detector, which is based on the same design libraries (Kraft *et al.*, 2009 ▶). However, the main deterioration is expected to come from the radiation-induced increase of leakage current in the silicon sensor.

**Figure 2:**
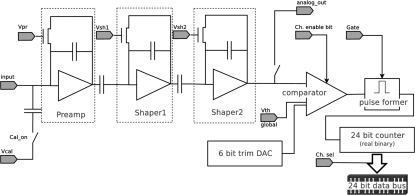
Schema of a channel of the MYTHEN readout ASIC.

Each channel of the ASIC is independent of the others and its architecture is sketched in Fig. 2 ▶. It consists of a charge-sensitive preamplifier AC-coupled to two shaping gain stages and followed by a comparator with adjustable threshold. Only the signals higher than the threshold are counted as photons by the internal 24-bits counter, thus rejecting the intrinsic electronic noise, as well as low-energy fluorescence photons. The counters are gateable, *i.e.* the time during which they count X-rays can be defined by an electronic digital signal which acts like a shutter. For this reason the use of the MYTHEN detector is also ideal for pump–probe measurements where the detector counts for very short user-defined time intervals.

The comparator threshold can be trimmed on a channel-by-channel basis by means of an internal 6-bit digital-to-analog converter (DAC) which adds to the global externally adjustable threshold. The parameters of the amplification and shaping chain can be externally regulated in order to optimize the noise and counting rate behavior. A calibration input allows the pulsing of the preamplifier input for test purposes; the analogue signal at the comparator entrance can also be measured for debugging. Malfunctioning channels can be individually disabled.

In addition, the chip contains the digital logic necessary to configure the internal DACs and read out the counters serially over four parallel data output lines. Since the detector cannot count while it is being read out, a partial readout of the counters is possible in order to reduce the readout time, although at the expenses of the dynamic range.

The digital signals are routed to a programmable logic chip (FPGA) which sends the control signals to the ASICs and returns the data to the acquisition system. The ASICs can be initialized individually, while the data acquisition is normally carried out in parallel for all chips.

The FPGA also controls the DACs for the adjustment of the amplification and shaping chain parameters, the amplitude of the trim-bits and the global comparator threshold, which are common to all of the ten ASICs hosted on a module.

### 2.3. The MYTHEN system

**Figure 3:**
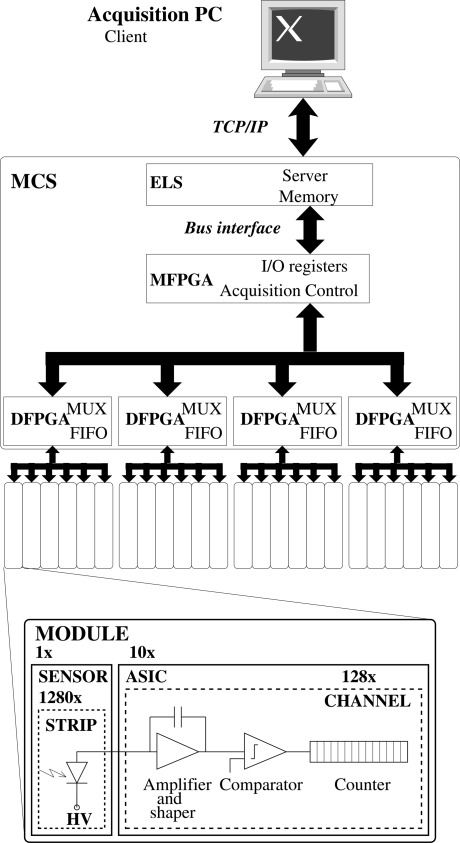
Sketch of the architecture of the MYTHEN detector.

A sketch of the architecture of the whole detector is shown in Fig. 3 ▶, where all the major components are indicated.

The MYTHEN control system (MCS) consists of a printed circuit board based on an embedded linux system (ELS) which controls five FPGAs operating the detector. The firmware has been developed in order to acquire data in real time.

The ELS runs at 100 MHz and has been chosen because of the flexibility and ease of implementation of the acquisition system. It communicates with the acquisition PC *via* a client-server architecture over a 100 Mbit Ethernet standard network. The maximum data transfer rate is about 4 MByte s^−1^ over TCP/IP.

The communication between the ELS and the main FPGA (MFPGA) is performed using the memory bus of the CPU running the embedded Linux system. The I/O registers implemented in the MFPGA are mapped to the ELS memory in order to achieve fast data transfer.

An application-specific state machine controls the acquisition and readout flow. The acquisition can be synchronized to external hardware by using an external digital gate signal defining the the time interval during which the detector is counting or a digital trigger signal to start the acquisition.

Four identical daughter FPGAs (DFPGAs) route the signals each to six individual modules. The DFPGAs multiplex the signal to the modules and contain FIFOs to store the data for four 24-bit frames or up to 32 4-bit frames. If the FIFOs cannot be read out fast enough by the ELS and become filled, the acquisition is stopped.

The time needed to read out all the ASICs in parallel is 250 µs for 24-bit dynamic range down to 90 µs for 4-bit partial readout. However, the maximum frame rate is limited by the data transfer rate over the network and can be as much as 10–100 frames s^−1^ (24–4 bits) for the whole 120° of the detector. In order to perform faster time-resolved measurements, it is also possible to transfer only the data from a limited number of modules, thus obtaining a maximum frame rate of 100–900 Hz (24–4 bits) for a single module (5° angular range).

## 3. Detector characterization

The MYTHEN detector has been used for many experiments at the SLS demonstrating the ability to return high-quality data (Olliges *et al.*, 2007 ▶; Nicula *et al.*, 2009 ▶; Cerny *et al.*, 2009 ▶). These results rely not only on the performance of the hardware but also on a very accurate calibration of the system.

### 3.1. Detector response

Single-photon-counting detectors are sensitive to single photons and the only limitation on the fluctuations of the number of counts is given by the Poisson-like statistics of the X-ray quanta. The digitized signal does not carry any information concerning the energy of the X-rays and all photons with an energy larger than the threshold are counted as one bit. This means that the choice of the correct comparator threshold level is critical in order to obtain good-quality data.

**Figure 4:**
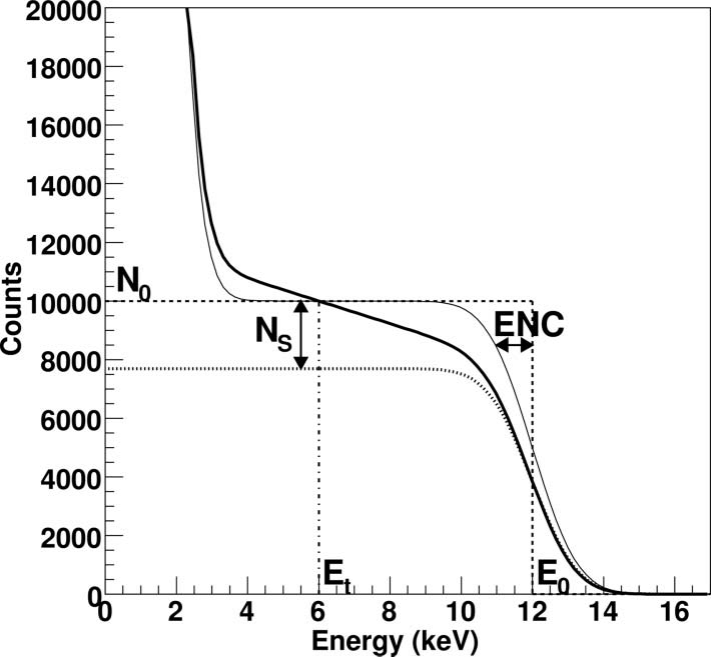
Expected counts as a function of a threshold energy for a monochromatic beam of energy *E*_0_ = 12 keV. *N*_0_ = 10000 is the number of photons absorbed by the detector during the acquisition time. The dashed line represents the curve in an ideal case without electronic noise and charge sharing, the solid thin line with noise ENC = 1 keV but without charge sharing, and the solid thick line is the physical case with noise and charge sharing *C*_s_ = 22%. *N*_S_ is the number of photons whose charge is shared between neighbouring strips (*C*_s_ = *N*_S_/*N*_0_). The dotted line represents the number of photons whose charge is completely collected by a single strip.

Fig. 4 ▶ shows the expected number of counts as a function of the threshold energy for *N*_0_ monochromatic X-rays of energy *E*_0_. This is often denominated the S-curve and can be interpreted as the integral of the signal spectrum between the threshold level and infinity. The dashed curve represents the behavior of an ideal counting system: nothing is counted for thresholds larger than the photon energy and all the *N*_0_ X-rays are counted for thresholds lower than *E*_0_. The thick solid line represents the physical curve which also takes into account the electronic noise and the charge sharing between channels.

The intrinsic noise on the electronic signal is defined by the equivalent noise charge (ENC) (Radeka, 1988 ▶). The ENC describes noise in terms of the charge at the detector input needed to create the same output at the end of the analog chain and is normally expressed in electrons. For silicon sensors, it can be converted into energy units by considering 1e^−^ = 3.6 eV. The value of the ENC normally depends on the shaping settings of the analog chain and increases with shorter shaping times. The resulting electronic signal spectrum is then given by a convolution between the radiation spectrum and the noise, *i.e.* a Gaussian of standard deviation ENC. The S-curve for a monochromatic radiation beam is well described by a Gaussian cumulative distribution *D* with an additional increase at low threshold owing to the baseline noise, as shown by the solid thin line.

Moreover, when a photon is absorbed in the region between two strips of the sensor, the generated charge is partially collected by the two nearest electronic channels. For this reason the physical S-curve is not flat but can be modeled by a decreasing straight line as described in detail by Bergamaschi *et al.* (2008 ▶) and Marchal (2010 ▶). The number of shared photons *N*_S_ is given by the difference between the number of counts and the number of X-rays whose charge is completely collected by the strip (shown by the dotted line).

The number of counts in the physical case is equal to that in the ideal case for a threshold set at half the photon energy. This defines the optimal threshold level *E*_t_ = *E*_0_/2.

The detector response *N* as a function of the threshold energy *E*_t_ is given by the sum of the noise counts *N*_n_ and the counts originating from photons *N*_γ_,
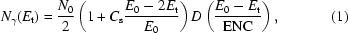
where *C*_s_ is the fraction of photons which produce a charge cloud which is shared between neighboring strips (*N*_s_ = *C*_s_ *N*_0_).

By assuming a noise of Gaussian type, and considering its bandwidth limited by the shaping time τ_s_, the number of noise counts in the acquisition time *T* can be approximated as
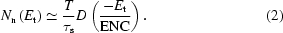
The choice of the comparator threshold level *E*_t_ influences not only the counting efficiency and noise performances but also the spatial resolution and the counting statistics of the detector. If the threshold is set at values higher than the ideal value *E*_t_ = *E*_0_/2, a fraction of the photons absorbed in the sensor in the region between two strips is not counted thus reducing the detector efficiency but improving its spatial resolution (narrower strip size). On the other hand, if the threshold is set at values lower than *E*_t_, part of the X-rays absorbed in the region between two strips are counted by both of them, resulting in a deterioration of the spatial resolution of the detector and of the fluctuations on the number of photons because of the increased multiplicity (Michel *et al.*, 2006 ▶).

### 3.2. Detector settings

Since the minimal detectable energy and the maximum count rate (see sections §3.3 and §3.4) depend both on the shaping time of the analog signal of the front-end electronics, three different settings have been defined for MYTHEN in order to cover a large range of applications by tuning τ_s_ and ENC (see Table 1 ▶):

*High-gain* settings are intended for applications where a low energy or a long acquisition time are required (small ENC) but the photon flux is limited (long τ_s_);

*Fast* settings are optimized for high count rates (short τ_s_) but can be used only at fairly high energies (large ENC);

*Standard* settings match most applications with regard to both the energy range and the count rate.

### 3.3. Minimum detectable energy

If the photon energy *E*_0_ of the X-rays is comparable with the electronic noise ENC for any chosen threshold level *E*_t_ < *E*_0_ there will be a non-negligible number of noise counts *N*_n_ and a loss of photon counts owing to the threshold value being too close to the photon energy.

A minimum threshold can be defined as the value Σ which is needed to obtain, on average, less than one noise count during the acquisition time *T*. From equation (2), with τ_s_ ≃ 0.5 µs and *T* = 1 s one obtains Σ ≃ 5 ENC. Σ should be adapted to the τ_s_ and ENC of the chosen detector settings and to the acquisition time *T* required.

For an optimal threshold set at half of the X-ray energy, this minimum threshold Σ corresponds to a minimum detectable energy of 2Σ ≃ 10 ENC.

**Figure 5:**
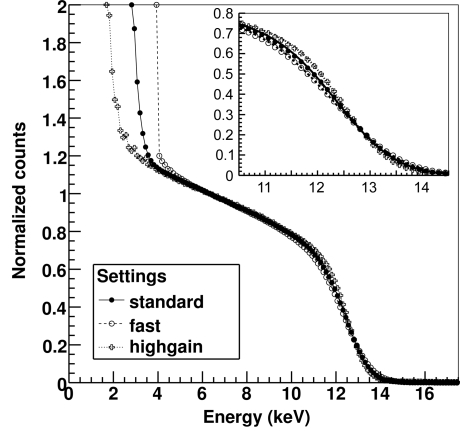
Measured threshold scan at 12.5 keV with the three different settings. In the inset the fit of the experimental data with the expected curve as in equation (1) is shown in the region of the inflection point.

Fig. 5 ▶ shows the average threshold scan of a channel of the detector obtained with the three different settings using X-rays of 12.5 keV. The fit to the model of equation (1) is shown in the inflection point region by the curves plotted in the inset, where the differences in the ENC values determine the steepness of the curve. The average ENC determined by the S-curve fit for the different settings on all the channels of the detector are ENC_standard_ = 0.83 ± 0.02 keV, ENC_fast_ = 0.94 ± 0.02 keV and ENC_high gain_ = 0.70 ± 0.02 keV, which results in a minimum detectable energy at 2Σ = 10 ENC of about 8 keV for the *standard* settings, 10 keV for the *fast* settings and 7 keV for the *high-gain* settings. For the *high-gain* settings it is still possible to measure 5 keV X-rays by setting the threshold at 3 keV at the cost of some efficiency loss and possibly some noise counts. The number of noise counts at lower thresholds is larger for the faster settings not only because of the increase in the ENC but also because of the shorter shaping time τ_s_ as expected from equation (2).

Although the presence of a minimum detectable photon energy is a disadvantage compared with integrating detectors, the improved dynamic range given by the possibility of detecting single photons and by the absence of saturation makes photon-counting systems optimal for experiments where small signals must be detected, *e.g.* for thin or weakly scattering samples.

The maximum detectable signal of a counting system is defined by the dynamic range of the counter, *i.e.* 24 bits in the case of MYTHEN. Since a photon-counting detector is readout-noise free, an even larger dynamic range can be achieved by summing separate frames without increasing the uncertainties.

### 3.4. Maximum count rate and rate corrections

In the case of photon-counting systems a deviation from the linearity on the number of counts occurs at high photon fluxes because of the pile up of the analog signal generated by the X-rays absorbed in a very short time in the same strip (Knoll, 1989 ▶; Leo, 1994 ▶). The loss of efficiency can be modeled for MYTHEN as for a paralizable detector,
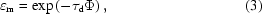
where Φ = *N*(*E*_t_)/*T* is the photon flux absorbed by the detector, and the dead-time τ_d_ is approximately the width of the signal at the threshold level and increases with the shaping time of the analog chain. τ_d_ places a maximum limit for the intensity of the beam above which it is impossible to correct for the loss of efficiency at Φ_max_ = τ_d_^−1^.

The efficiency of the detector as a function of the count rate has been calibrated according to equation (3). The dead-time τ_d_ was estimated by acquiring the diffraction pattern from a silicon powder with varying beam intensities and calculating the value that best corrects the data acquired under high photon fluxes. In order to avoid fluctuations between the measurements, the silicon capillary and the detector were kept stationary with the beam matching the sample size. The beam was attenuated by means of aluminium filters of different thicknesses installed at the MS beamline. The attenuation of the filters was estimated by integrating the number of counts over the background regions of the silicon powder pattern acquired. This method is able to provide the necessary dynamic range and intensity resolution, with the further advantage of intrinsically monitoring the intensity of the beam and mainly discarding the higher harmonics components of the beam, given their low elastic scattering cross section and low absorption efficiency in the sensor.

The measurements were performed with the standard filling pattern of the SLS, which consists of a flat-filled electron beam of 780 ns with 390 electron bunches of approximately 20 ps length every 2 ns followed by a gap of 180 ns. The estimated τ_d_ will be approximately the same also with the other filling mode of the SLS, *i.e.* with an additional electron bucket filled in the gap.

The measurements have been performed at the energy which provides the maximum photon flux, *i.e.* *E*_0_ = 12.4 keV, and the maximum number of counts of the Si(111) peak during the measurements ranged between 10^3^ and 3 × 10^6^ counts s^−1^. The average τ_d_ values estimated for the pre-defined settings over several channels for the detector are τ_standard_ = 170 ± 10 ns, τ_fast_ = 110 ± 10 ns and τ_high gain_ = 750 ± 50 ns for the threshold set at half of the X-ray photon energy. τ_d_ is inversely related to the threshold level. However, small differences between channels, threshold values or photon energies are not significant since ∊_m_ is only weakly dependent on the value of τ_d_.

**Figure 6:**
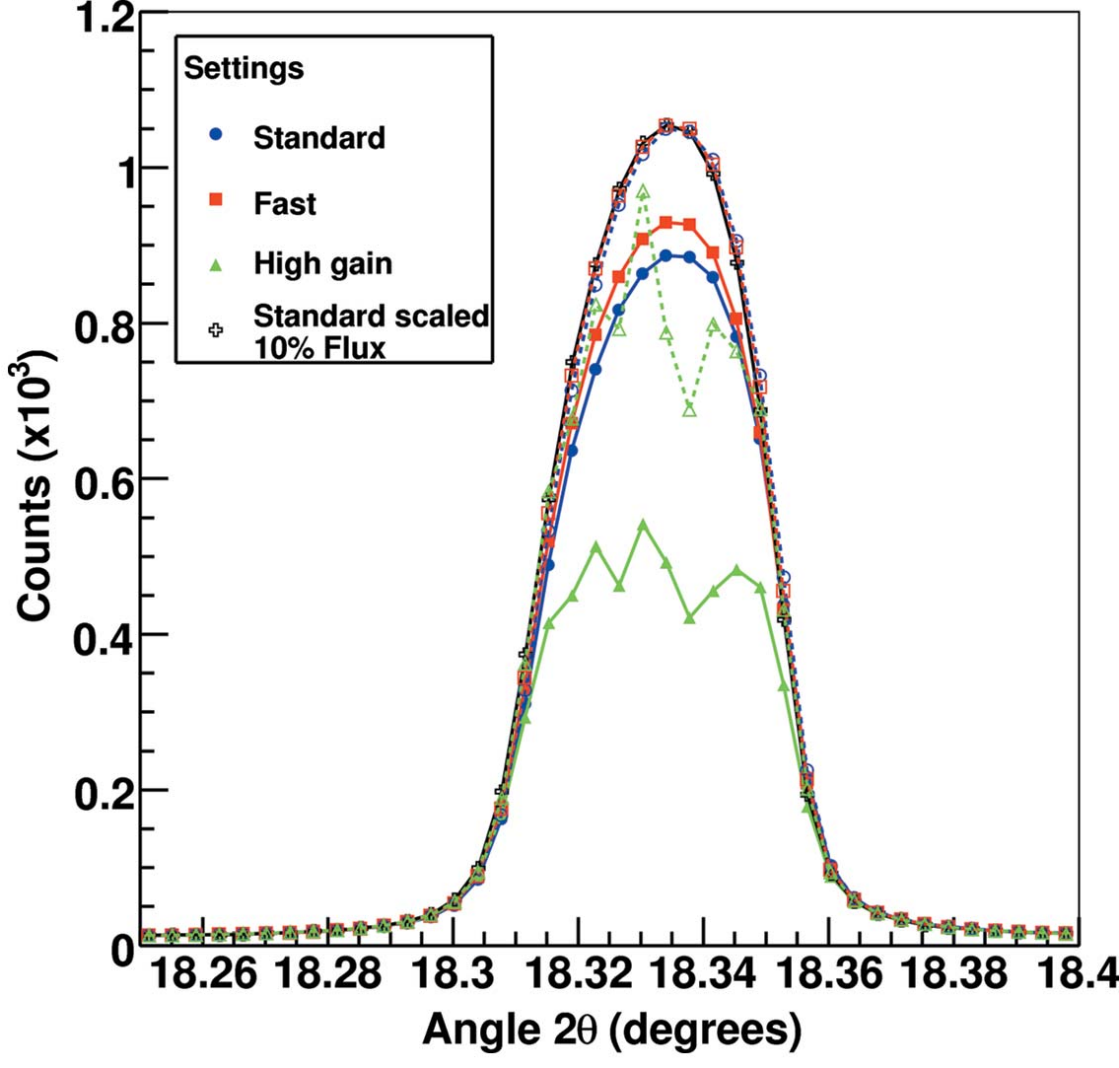
The Si(111) peak measured without any attenuation of the beam at 12.4 keV using *standard*, *fast* and *high-gain* settings compared with a measurement at low flux rescaled to compensate for the beam attenuation. The solid line with filled markers refers to the measured data, while the dashed line with empty markers represents the rate-corrected data points using the values listed in Table 1 ▶. The *high-gain* settings are not corrected close to the peak maximum since the count rate is larger than Φ_max_.

Fig. 6 ▶ shows the Si(111) peak measured in 1 s at 12.4 keV without any attenuation using the different settings before and after applying a rate correction to the data according to equation (3), compared with a measurement at low flux rescaled to compensate for the beam attenuation. The corrected data properly match the low-intensity measurement for all count rates (*i.e.* different positions on the peak) except in the case of the high-gain settings when the count rate is larger than the estimated Φ_max_ (*i.e.* close to the peak maximum), as expected.

### 3.5. Threshold calibration and equalization

The choice of the level of the comparator threshold plays a very important role in counting systems since it influences the efficiency of the detector as well as its spatial resolution, as described in §3.1.

**Figure 7:**
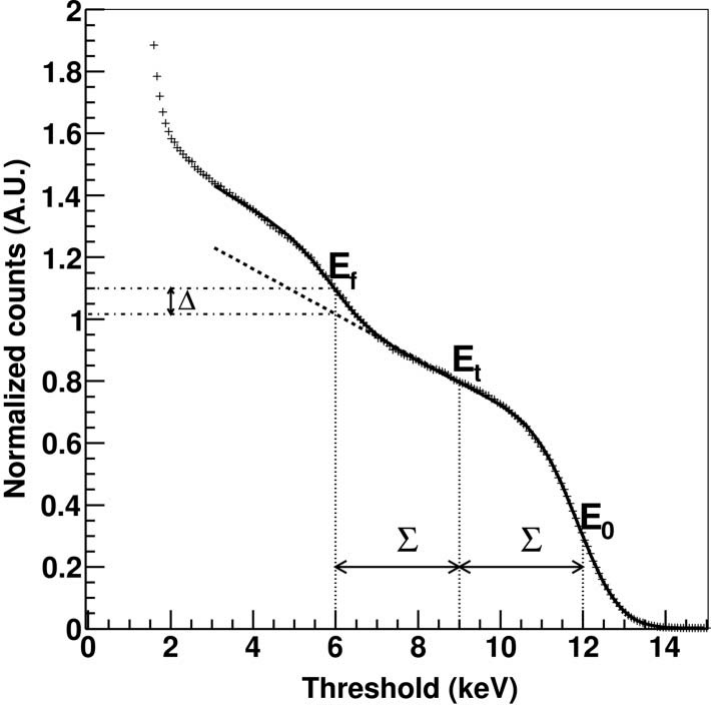
Number of counts as a function of the threshold measured from a sample containing iron (*E*_f_ = 5.9 keV) when using X-rays of energy *E*_0_ = 12 keV. In this case, setting the threshold at *E*_0_/2, which is very close to *E*_f_, would give Δ ≃ 10% counts from the fluorescence background. Therefore the threshold should be set at an intermediate level *E*_t_ between the two energy components with a distance of at least Σ > 3 ENC from both *E*_f_ and *E*_0_.

Furthermore, the threshold uniformity is particularly critical with regards to fluorescent radiation emitted by the sample under investigation. Since the emission of fluorescent light is isotropic, the data quality will be improved by setting the threshold high enough in order to discard the fluorescence background (see Fig. 7 ▶). Moreover, setting the threshold too close to the energy of the fluorescent light gives rise to large fluctuations between channels in the number of counts since the threshold sits on the steepest part of the threshold scan curve for the fluorescent background. These differences cannot be corrected by using a flat-field normalization (see section §3.6) since the fluorescent component is not present in the reference image. For this reason it is extremely important that the threshold uniformity over the whole detector is optimized. The threshold level must be set at least Σ > 3 ENC away from both the fluorescent energy level and the X-ray energy in order to remove the fluorescence background while efficiently count the diffracted photons.

The comparator threshold is given by a global level which can be set on a module basis and adds to a component which is individually adjustable for each channel. In order to optimize the uniformity of the detector response it is important to properly adjust the threshold for all channels.

**Figure 8:**
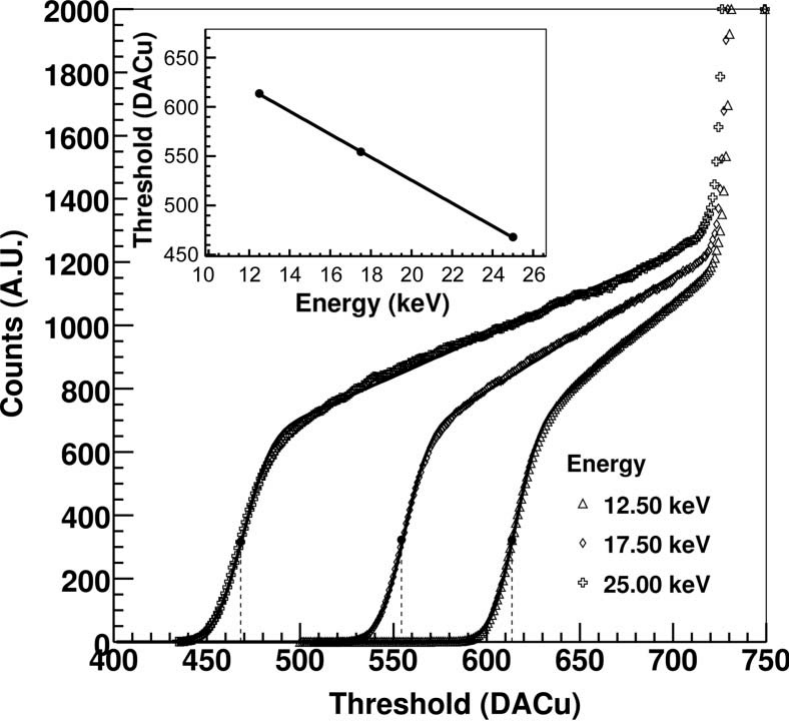
Median of the number of counts as a function of the threshold for X-rays of 12.5, 17.5 and 25 keV for one of the detector modules using *standard* settings. The solid line represents the fit of the experimental points with equation (1). In the inset the linear fit between the X-ray energy and the position of the inflection point of the curves is shown.

Since both the signal amplification stages and the comparator are linear, it is necessary to calibrate the detector offset *O* and gain *G* in order to correctly set its comparator threshold *V*_t_ at the desired energy *E*_t_: 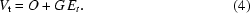
This is initially performed by acquiring measurements while scanning the global threshold using different X-ray energies and calculating the median of the counts at each threshold value for each module *i*. The curves obtained for one of the detector modules at three energies are shown in Fig. 8 ▶. The experimental data are then fitted according to equation (1) and for each module a linear relation is found between the X-ray energy and the estimated inflection point, as shown in the inset of Fig. 8 ▶. The resulting offset *O*_i_ and gain *G*_*i*_ are used as a conversion factor between the threshold level and the energy.

Differences in gain and offset are present also between individual channels within a module and therefore the use of threshold equalization techniques (trimming) using the internal 6-bit DAC is needed in order to reduce the threshold dispersion (Bergamaschi *et al.*, 2009 ▶). Since both gain and offset have variations between channels, the optimal trimming should be performed as a function of the threshold energy.

The detector is initially trimmed without X-rays by assuming a constant electronic noise on the whole detector. This basically consists of compensating for the offset differences between channels and improves the threshold dispersion by a factor of more than seven compared with the untrimmed case.

The trimming is then improved by compensating also for the gain difference between channels by adjusting the thresholds of the single channels in order to obtain a uniform number of counts over the whole detector. Since a uniform illumination of the detector is required, it can only be obtained by scanning the system at a constant velocity in front of the radiation scattered at wide angle by an amorphous material and takes about 45 min, limited by the rotation speed of the detector arm. The differences in the number of counts owing to threshold mismatches are enhanced by the steepness of the S-curve when using photons of the same energy as the threshold, and the fluctuations owing to, for example, efficiency differences or beam stability can be neglected.

**Figure 9:**
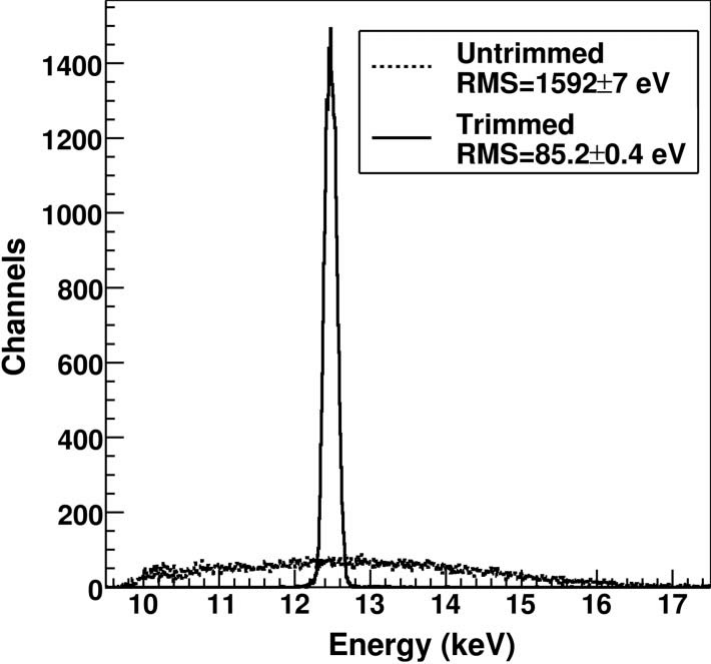
Threshold dispersion over the whole detector at 12.5 keV in the untrimmed and trimmed case with *standard* settings.

Fig. 9 ▶ shows the threshold dispersion obtained over the whole detector at 12.5 keV with *standard* settings before and after an optimization of the trimming. The improvement of the threshold dispersion owing to trimming is almost a factor of 15.

Table 2 ▶ lists the threshold dispersions measured for various X-ray energies and detectors settings. The threshold dispersion increases with the photon energy, since the non-uniformities owing to gain mismatches are more visible at high energies and, for the same reason, it is generally larger when using *high-gain* settings.

### 3.6. Flat-field correction

The fluctuations in the comparator threshold level between channels cause differences in the number of counts. Considering only the differences owing to the charge sharing as discussed in §3.1, a threshold dispersion of 100 eV with the threshold set at 6 keV corresponds to variation of counts between channels owing to the threshold fluctuations of approximately 1% for 12 keV photons. This value exceeds the fluctuations owing to the Poisson-like statistics only when more than 10000 photons per channel are detected.

An average value of 1.1 ± 0.7% relative fluctuations on the number of counts has been measured for the single modules, which is close to the above estimate. This value is not only due to the threshold dispersion but also to other fluctuations, *e.g.* the efficiency of the sensor.

Larger fluctuations over the number of counts on the whole detector (5.93 ± 0.09%) are, however, due to the uncertainties in the threshold calibration of the modules and to the geometrical variations in the size of the entrance window of the detector owing to mechanical deformations of the detector housing around its center.

For all these reasons it is mandatory to apply a flat-field correction consisting of normalizing the data using an image acquired with a uniform illumination at the working X-ray photon and threshold energies.

In order to uniformly illuminate all the channels, the detector is translated at a constant velocity in front of the beam scattered at wide angle by an amorphous material (*e.g.* a silica rod). The movement is repeated several times in order to accumulate statistics and average out possible fluctuations of the beam during the acquisition and various systematic errors given by the detector movement (*e.g.* effect of gravity, variations of the rotation speed). The statistics of the flat-field data need to be sufficiently high in order to give a negligible contribution to the statistical error of the measurement data. Typically 10^5^–10^6^ counts per channel are acquired for flat-field corrections and the procedure can take from half an hour up to several hours depending on the required statistics and on the rotation speed of the diffractometer. However, the acquisition of the flat-field data corresponding to the same photon and threshold energies needs to be repeated only occasionally.

### 3.7. Bad channels

On average the number of bad channels which are either too noisy (hot channels) or blind to X-rays (dead channels) is about two per module, *i.e.* less than 0.2% of the total. The bad channels of the detector are listed in a file and their readout value is completely discarded in the data processing without interpolation.

The compromise between low threshold dispersion and the ability to trim all channels leads to channels whose individual thresholds are outside of the dynamic range of the trimming. Hot channels have an effective threshold which is lower than that of the rest of the module and therefore the number of noise counts is not negligible. Channels with an effective high threshold and thus a reduced efficiency can normally be corrected by flat-field normalization.

The main reason for the presence of dead channels is faulty wirebonds between the ASIC and the sensor. The neighbors of a dead channel additionally detect part of the X-rays absorbed in the floating strip leading to an excess of counts that can also be corrected by flat-field normalization.

### 3.8. Angular calibration

In order to convert from strip number to 2θ angle, an accurate angular calibration of the detector must be performed. For this purpose a series of patterns of a silicon powder are acquired while shifting the detector by 0.1°.

In a first step, the Si(111) peak is fitted with a Gaussian in order to determine its position *C*_peak_ in channel number for each of the acquired patterns.

In a second step, for each module *i* the encoder position Θ_e_ is fitted as a function of the peak position *C*_peak_ according to
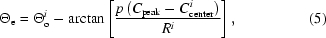
where the parameter 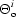 is the angular offset with respect to the diffractometer zero position, 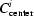 is the central channel and *R* ^*i*^ is the distance of the module *i* from the diffractometer center while *p* = 50 µm is the strip pitch of the detector.

Finally, the global offset of the detector system is precisely determined by refining a silicon pattern at a well defined energy [*i.e.* knowing the position of the Si(111) peak].

The same function as equation (5), with the parameters obtained from the calibration, is used in order to convert from channel number to 2θ angle.

The parallax at the borders of the modules owing to the thickness of the silicon sensor is a function of the X-ray energy (higher-energy X-rays are absorbed deeper inside the sensor) and is of the order of 0.2 mdeg at 12 keV and 0.5 mdeg at 30 keV.

The differences in pixel size owing to the different portion of solid angle covered by the strips on the border of the modules and the higher efficiency owing to the longer path of the X-rays in the sensor are removed by the flat-field correction. This also normalizes additional differences in pixel size between channels which are also present because of mis­matches in the strip sensor fabrication and in fluctuations of the channels threshold level as discussed at the end of §3.1.

Patterns acquired at different detector positions are generally merged together in order to fill the gaps between the modules and correct possibly bad functioning channels. In this procedure the data from different positions which are closer than 4 mdeg (the average pixel size) are averaged and the new position is set to the mean of the positions of the original points.

The position and width of the peaks result from a fit over several detector channels. Geometrical distortions might disturb this determination mainly because of errors in the angular calibration, fluctuations in the encoder position, variations between channels and parallax effects.

The resolution in locating the peak center and determining its width and integrated intensity has been estimated by acquiring several patterns of a LaB_6_ sample in a 300 µm capillary with the detector shifted in 5 mdeg steps between 30.4 and 36.5°. The 16 peaks acquired have been fitted with a Gaussian function plus background and the fluctuations on the fitted parameters have been calculated. The resulting average resolutions are 0.63 ± 0.06 mdeg for the peak center and 0.22 ± 0.05 mdeg for the peak FWHM for an average peak FWHM of 27.0 ± 2.5 mdeg.

These results show that the angular calibration allows a resolution in determining the peaks position and width which is appropriate for structural determination.

### 3.9. Bragg peak angular resolution

The Bragg peaks diffracted by polycrystalline samples have a finite width which limits the possibility of separating peaks very close to each other. This broadening originates from the sample microstructure, from the contributions of all the optical elements in the beam path and from the detector. Knowledge of the instrumental contributions to broadening is important in order to evaluate the absolute resolving power of the instrument and, when needed, to subtract this contribution for the evaluation of the sample-intrinsic broadening, which is often the main object of study.

The instrumental broadening is the convolution of the broadening owing to the optics chain before the sample and of the sample geometrical broadening which, in the absence of a diffracted beam analyzer, is caused by the sample size and by the strip pitch.

The broadening owing to the sample microstructure is almost always well represented by a Voigt function, *i.e.* a convolution of a Gaussian and a Lorentzian, while the broadening arising from the optics and from the sample geometry are well approximated by pure Gaussian profiles. Therefore in the following only the Gaussian widths are considered, which are well represented by their standard deviations.

The total peak broadening σ is given by the quadratic sum of the microstructural broadening σ_sample_ and of the instrumental broadening,
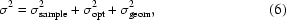
where σ_opt_ is the broadening owing to the optics chain before the sample and σ_geom_ represents the sample geometric broadening.

The contribution σ_opt_ introduced by the optics chain arises mainly from beam divergence and depends on the scattering angle 2θ as follows (Caglioti *et al.*, 1958 ▶; Gozzo *et al.*, 2006 ▶), 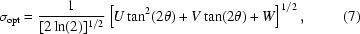
where the units are degrees, *U*, *V* and *W* are the Caglioti half-width parameters, and the numeric factor 1/[2ln2]^1/2^ converts the half-width at half-maximum of a Gaussian to its standard deviation.

Hereafter, the instrumental contribution σ_geom_ to the Bragg peak broadening when using a MYTHEN detector is discussed in the case of samples delimited by a cylindrical container (glass capillary) rotating around their axis normal to the diffraction plane in order to increase the powder orientational isotropy (Debye–Scherrer geometry).

σ_geom_ can be separated into four main contributions which can be modeled by their Gaussian standard deviations and quadratically summed,
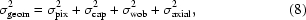
where:

σ_pix_ is due to the detector intrinsic resolution and is mainly defined by the strip pitch of the sensor. The point spread function of MYTHEN has been measured by Bergamaschi *et al.* (2008 ▶) and has a standard deviation σ_pix_ = 16 ± 2 µm, which differs from the ideal standard deviation of a 50 µm wide box function (14.4 µm) because of the presence of charge sharing between neighboring strips, as explained in §3.1. This can be converted into 2θ angle using the sample-to-detector distance *R* = 760 mm and results in σ_pix_ = 1.21 ± 0.15 mdeg. These values have been measured with an X-ray photon energy of 8 keV and threshold set at 4 keV. σ_pix_ is inversely related to the threshold value, while its weak relation with the X-ray photon energy can be neglected since the average absorption depth is about half of the wafer thickness for all energies larger than 8 keV.

σ_cap_ is due to the finite and usually not negligible diameter of the capillary. If the effects of absorption in the sample are small, which can be obtained by adjusting the loading density so that the X-ray absorption depth is larger than the capillary diameter, the contribution arising from the size of the capillary can be calculated by considering the projection of the cylindrical capillary on the flat surface of the detector,
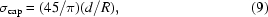
where σ_cap_ is expressed in degrees, *d* is the sample diameter and *R* is the sample-to-detector distance. σ_cap_ linearly spans from 2 mdeg for a 0.1 mm capillary up to 19 mdeg for a 1 mm capillary.

σ_wob_ is due to the possible misalignment between the capillary axis and its rotation axis (wobbling) and can be evaluated by convolving the cylindrical capillary shape with the periodic wobbling function and averaging its projection on the flat detector surface,
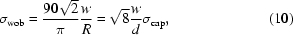
where *w* is the displacement of the capillary. For *w* ≃ 0.1*d* the contribution to the broadening is ∼4% of the total sample geometrical contribution σ_geom_. Since it is usually possible to align capillaries to within 0.05 mm or better, medium capillary sizes are less affected by wobbling, although lower sizes may have a significant wobbling contribution. If wobbling is severe (*w* > 0.5*d*) the peak shape changes significantly, even becoming bimodal, and standard analysis software cannot easily cope with this case. Therefore, a good capillary axial alignment is essential, at least when the intrinsic microstructural broadening is not large.

σ_axial_ is due to the detector and sample axial dimension, known as the Finger–Cox–Jephcoat (FCJ) lineshape (Finger *et al.*, 1994 ▶), whose contribution σ_axial_ is proportional to cot(θ). With an axial dimension of 8 mm given by the MYTHEN strip length, σ_axial_ can be considered negligible for 2θ > 10°. Magnitudes of corrections to the various moments can be easiest computed according to formulas found by Prince & Toby (2005 ▶).

Several patterns of silicon powder capillaries have been collected at different energies (from 8 keV to 28 keV) in a broad angular range in order to measure the Bragg peak broadening. Although silicon is not the usual choice as a line profile standard, since sample microstructural broadening effects are not negligible, it has been chosen because of its relatively low absorption coefficient in order to examine a broad energy spectrum and a large range of capillary diameters (0.2 mm to 1 mm), as commonly used in experiments. For the largest diameters and the lowest energies, even pure silicon powder was not satisfactory and it has been diluted with a reasonable proportion of amorphous light-element glass powder, so as to decrease the effective loading density and to increase the X-ray penetration length without compromising the homogeneity. Still, a strong absorption was visible with thicker capillaries at the lower energies (*d* > 1 mm at 12.4 keV and *d* > 0.8 mm at 8 keV).

**Figure 10:**
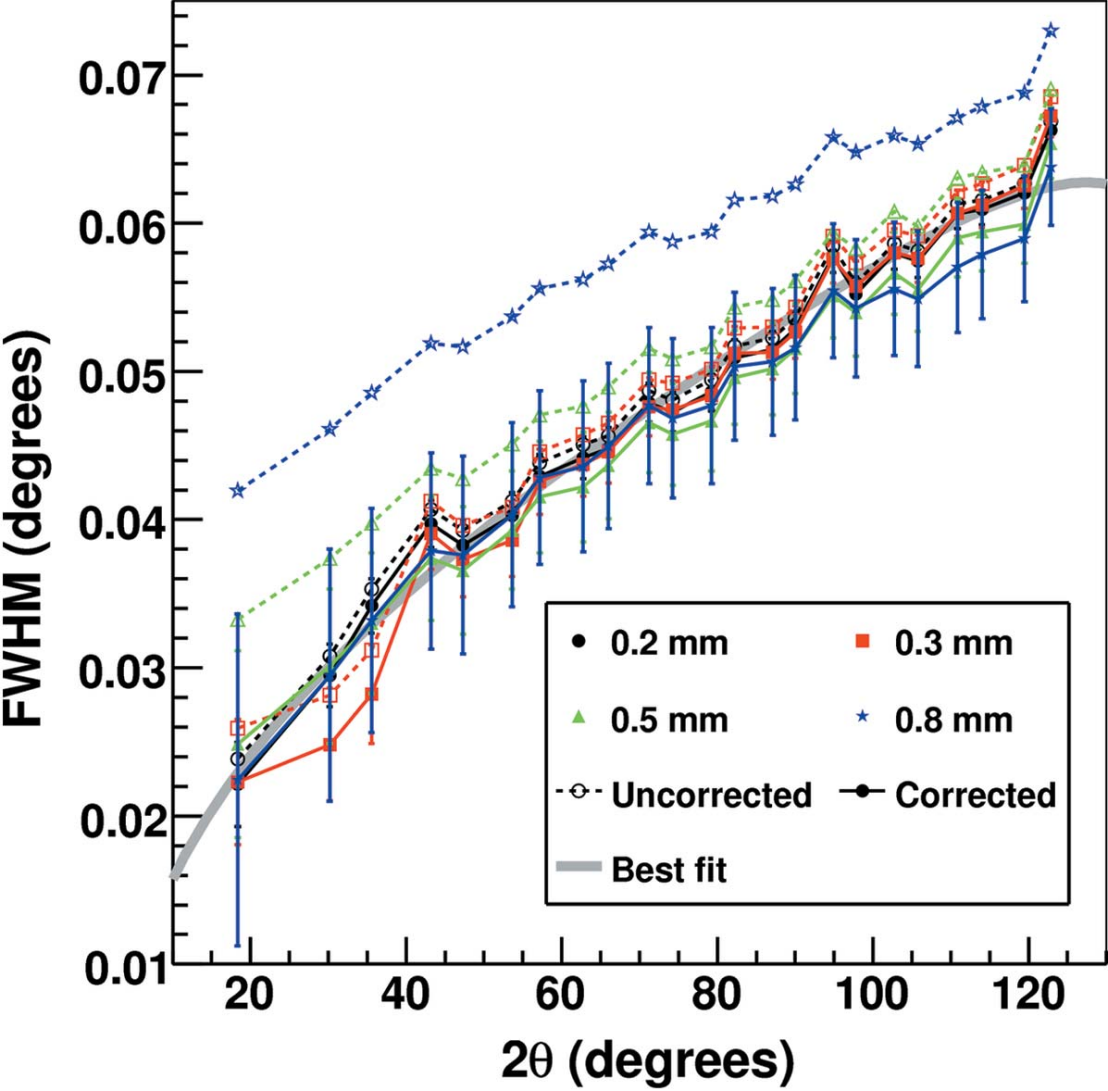
FWHM of Si peaks plotted *versus* 2θ for capillaries of different diameters for patterns collected at 12.4 keV obtained by Voigt profile fits. The measured (uncorrected) FWHMs are represented by the empty markers connected by a dashed line, while the corrected FWHMs are drawn as filled markers connected by a solid line. The gray solid line represents the best fit to the corrected values as from equation (7). The corrected FWHMs are calculated by subtracting the geometrical contribution σ_geom_ from the total peak broadening σ as from equation (6) using FWHM = 2[2ln2]^1/2^σ. The assumed wobbling radius 0 ± 20 µm is the dominant contribution to the error bars shown.

Special care has been taken to align the capillaries along the spinning axis using a microscope. The wobbling radius *w* was estimated to be below 20 µm. Fig. 10 ▶ shows the width of the Bragg peaks of silicon acquired at 12.4 keV as a function of the 2θ angle before and after subtracting the geometrical contribution as from equation (6). Similar results have also been obtained at 8 keV and 28 keV.

After the subtraction of σ_geom_, the peak variances relative to the different capillary diameters fall satisfactorily on the same curves. Although even anisotropic sample contributions are clearly present, it is demonstrated that the sample geometric contribution can be correctly determined and eliminated in order to evaluate the contribution owing to the microstructural properties of the sample.

## 4. Experimental results

In this section the quality of the data acquired using the MYTHEN detector installed at the SLS XRPD station is evaluated. For this purpose the performances are checked against whole powder pattern fittings, using both whole powder pattern matching (WPPM) (LeBail *et al.*, 1988 ▶; Pawley, 1981 ▶; Toraya, 1986 ▶) and structural refinements (Rietveld, 1969 ▶) of standard samples.

The advantage of parallel and fast acquisition of full diffraction patterns is also highlighted in relation to the phenomenon of radiation damage of organic compounds.

### 4.1. Standard powder samples

Whole diffraction patterns from the certified silicon powder from the National Institute of Standards and Technology (Si NIST 640C) and the fluoride Na_2_Ca_3_Al_2_F_14_ (Courbion & Ferey, 1988 ▶), denoted NAC hereafter, were refined using the *FullProf* program (Rodriguez-Carvajal, 1993 ▶).

All samples were mounted in Lindemann capillaries spinning at 10 Hz and measured in Debye–Scherrer (transmission) geometry. The patterns were least-squares fitted using a modified pseudo-Voigt profile (Thompson *et al.*, 1987 ▶) convoluted with the FCJ asymmetry function to model the low-angle peak asymmetry owing to axial divergence (Npr = 7 *FullProf* flag). The goodness-of-fit (GoF) indicator (McCusker *et al.*, 1999 ▶) is used to assess the quality of the refinements.

The accurate estimate of the working wavelength λ, the 2θ zero-offset and the profile parameters for both Si NIST 640C and NAC refinements as returned by *FullProf* are listed in Table 3 ▶.

Si NIST 640C has been chosen since it is a well defined NIST standard and therefore it is normally the preferred choice for the determination of the photon wavelength λ and 2θ zero-offset prior to measurements.

**Figure 11:**
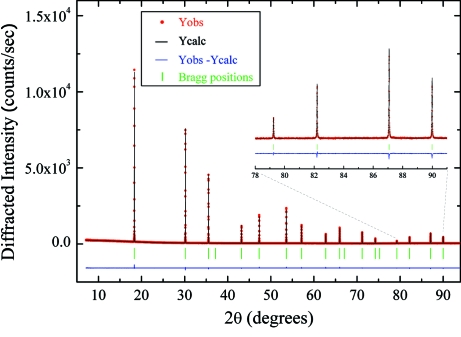
Powder pattern structure refinement of Si NIST 640C in a 0.5 mm capillary performed with *FullProf* at 12.4 keV photon energy.

The XRPD full diffraction pattern of a 0.5 mm capillary filled with Si NIST 640C measured at 12.4 keV is shown in Fig. 11 ▶. Multiple patterns were recorded at different detector positions for a total acquisition time of 2.4 s. A whole pattern structural refinement has been performed using the NIST reference values for the crystal structure resulting in a GoF = 1.4.

NAC has been chosen because of its small intrinsic line width and, therefore, it is appropriate for studying the instrumental contribution to the diffraction peak broadening for capillaries of the same size (see §3.9).

**Figure 12:**
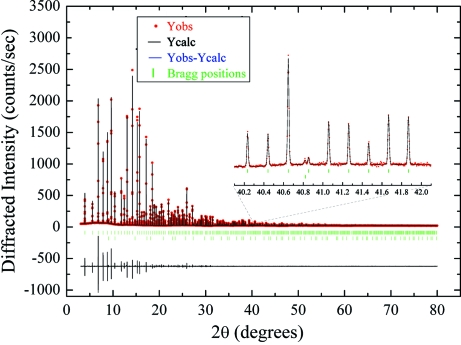
WPPM of Na_2_Ca_3_Al_2_F_14_ (NAC) in a 0.2 mm capillary performed with *FullProf* at 25 keV photon energy.

A full diffraction pattern collected in transmission at 25 keV of a NAC powder mounted in a 0.2 mm capillary is shown in Fig. 12 ▶. Multiple patterns were collected at different detector positions for a total acquisition time of 120 s.

Since NAC is not available as a NIST standard reference material, there are no certified values for lattice parameters, average grain size and residual strain. Therefore, the NAC lattice parameters and structure were taken from the literature (Courbion & Ferey, 1988 ▶). The grain size of the NAC powder used has been estimated at approximately 1.3–1.4 µm (Gozzo *et al.*, 2006 ▶).

In order to accurately determine the instrumental resolution function (IRF), a WPPM was performed with a resulting GoF = 1.1. The inset in Fig. 12 ▶ shows how clearly the reflection from the main NAC phase and from a known impurity (CaF_2_) are detected and distinguished even when they correspond to very close *d*-spacings.

The larger value of residuals (*Y*_obs_ − *Y*_calc_) characterizing the low 2θ-angles (<10°) of the NAC refinement is due to a mismatch between the observed and calculated 2θ value by up to half a strip (<0.002°) which defines the current limit of the MYTHEN angular calibration as described in §3.8. This effect is negligible for the majority of samples under investigation, which are normally characterized by intrinsic peak widths larger than NAC.

**Figure 13:**
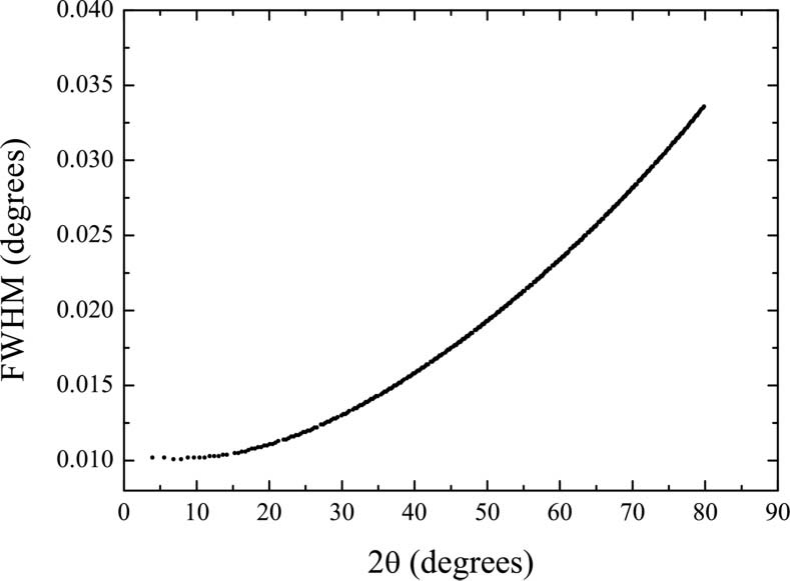
Experimental IRF as determined with a NAC powder in a 0.2 mm Lindemann capillary at 25 keV photon energy.

Fig. 13 ▶ shows the IRF (FWHM *versus* 2θ) measured using NAC in a 0.2 mm capillary at 25 keV.

### 4.2. Monitoring of the effect of radiation damage on organic compounds

Radiation damage of organic materials owing to their exposure to intense synchrotron beams often undermines the success of a structural solution (Holton, 2009 ▶). This is a very well known effect in macromolecular crystallography; it causes the disappearing of the high *d*-spacing resolution data, induces disorder in the heavy-atom sites in anomalous dispersion measurements and stimulates chemical changes of the molecules (Holton, 2007 ▶; Oliéric *et al.*, 2007 ▶; Schiltz *et al.*, 2004 ▶), and is often at the origin of unsuccessful structural solutions and/or of depiction of incorrect conclusions about the molecular functionality.

Low-temperature data acquisition reduces the effects of radiation damage, but the problem is still serious, in particular at third-generation synchrotron facilities.

Small organic molecular compounds (*e.g.* pharmaceuticals) are also strongly affected by radiation damage. In this case the structural solution *via* efficient high-resolution XRPD with one- or two-dimensional detectors, preferably at low temperature, can be an attractive viable alternative to single-crystal X-ray diffraction, because it does not need the acquisition of multiple data sets as in the case of single-crystal X-ray diffraction and data acquisition can therefore be extremely fast.

Furthermore, the recent development of direct-space structural solution methods that exploit the chemical knowledge of the molecule have dramatically increased the rate of success of *ab initio* structure solution (Harris *et al.*, 2004 ▶; Shankland *et al.*, 1998 ▶). Recently, Margiolaki & Wright (2008 ▶) successfully extended the use of powder diffraction to the study of proteins.

Structural solutions of organic compounds from synchrotron high-resolution data have been achieved by using multicrystal analyzer detectors and a data collection strategy that implies the transverse motion of a long capillary with respect to the incident beam to progressively expose fresh portions of the sample to the beam while the 2θ scan is being completed (Margiolaki *et al.*, 2005 ▶; Seijas *et al.*, 2009 ▶). In spite of having produced several successful structural solutions, this strategy has the potential danger of not being able to exactly identify when in the 2θ pattern the powder has overcome an irreversible damage.

MYTHEN provides the FWHM and *d*-spacing resolution required for the structural solution of organic compounds, with the further advantage of an outstanding counting efficiency and simultaneous acquisition of the full diffraction pattern.

**Figure 14 fig14:**
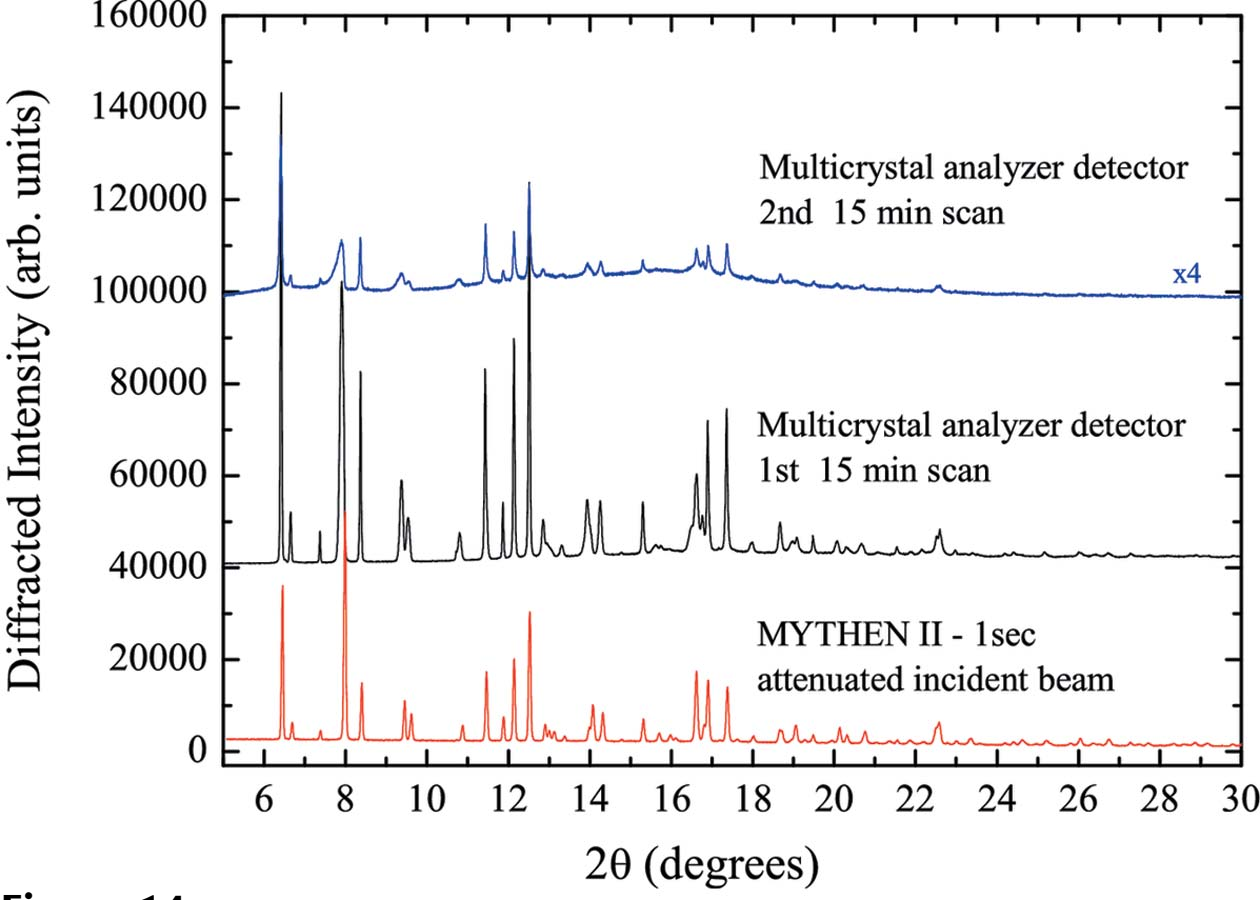
Full powder diffraction patterns of form D of bupivacaine hydrochloride in 1.0 mm Lindemann capillaries at 12 keV as collected with the multicrystal analyzer detector for 15 min twice (top and middle patterns) and MYTHEN in 1 s (bottom).

Diffraction patterns of bupivacaine hydrochloride, a long-acting anesthetic drug of the amide type used for local anesthesia (Niederwanger *et al.*, 2009 ▶), were part of a study of the polymorphism of bupivacaine, which is still in progress (Gozzo *et al.*, 2010 ▶). Fig. 14 ▶ shows, as an example, the powder diffraction patterns of form D of bupivacaine hydrochloride collected at the SLS powder diffraction station using the Si(111) multicrystal analyzer detector and MYTHEN at 50% reduced beam intensity in a 1 mm Lindemann capillary at 12 keV.

The first and second powder diffraction patterns collected using the multicrystal analyzer detector were acquired for 15 min by continuously moving the detector arm between 0° and 50° in 2θ whereas several 1 s diffraction patterns were collected with MYTHEN covering simultaneously 120° in 2θ. A comparison of the first and second multicrystal analyzer diffraction patterns clearly shows that the first 15 min of measurement already clearly damaged the sample.

**Figure 15:**
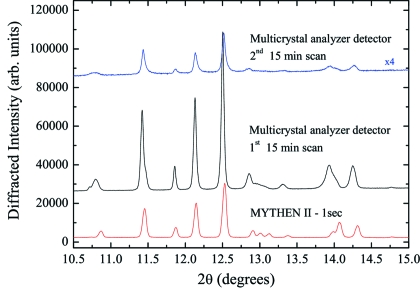
Detail of the diffraction patterns in Fig. 14 showing the effect of radiation damage on the bupivacaine organic powder.

A careful inspection of the first diffraction pattern and the comparison with the pattern collected in 1 s with MYTHEN (see Fig. 15 ▶) clearly show that the effect of damage was not negligible even in the first pattern after the acquisition of the first 10°. The structures at 13° and 14° are already strongly compromised by the radiation damage. Successive multiple 1 s data acquisitions with MYTHEN, always at reduced intensity, showed that the powder was stable for at least 2–3 min. While the data collected with the multicrystal analyzer detector were not usable for indexation and structural solution, MYTHEN data brought a successful structural solution of form D of bupivacaine in *P*2_1_2_1_2_1_ space group type.

The possibility of acquiring large statistics in short acquisition times opens the way towards *in situ* measurements of radiation-sensitive samples.

## 5. Conclusions and perspectives

The microstrip detector installed at the Material Science beamline of the Swiss Light Source is a unique instrument for powder diffraction experiments over a large angular range thanks to its outstanding characteristics and to the optimized calibration protocol. MYTHEN is increasingly utilized by the powder diffraction users at the SLS not only for time-resolved experiments, for which it was initially designed, but also for structural determination and refinement and for pair distribution function measurements.

With a proper set-up of the beamline optics and thanks to a careful calibration of the detector and diffractometer, high-quality powder diffraction data were acquired with the MYTHEN detector with the advantage of measurement times 5000–15000 times faster than using the crystal analyzer detector, depending on the X-ray energy and *d*-spacing resolution required.

It has been shown that the possibility of acquiring the diffraction pattern simultaneously on the whole angular range allows the acquisition of sufficient statistics before radiation damage occurs to the samples and the monitoring of the structural modifications over time, resulting in a powerful tool for the study of organic molecular compounds, *e.g.* for pharmaceutical applications.

Moreover, time-resolved studies (Fadenberger *et al.*, 2010 ▶) impossible with any other powder diffraction detector with the same angular range and angular resolution (FWHM) can be performed, opening new perspectives for *in situ* XRPD.

The modularity of the detector also allows for simultaneous control of detector systems composed of several independent parts. For example, a set-up for measurements in the horizontal diffraction plane is also available to investigate anisotropic effects (*e.g.* stress–strain) (Olliges *et al.*, 2007 ▶). An additional detector module for small-angle scattering measurements mounted on the wall of the experimental hutch at approximately 2 m from the sample allows, for example, the simultaneous monitoring of the nucleation and crystallization processes occurring in the samples.

A major upgrade of the readout system is foreseen in the second half of 2010 to further improve the frame rate capability of the detector by adding a larger memory on the acquisition board in order to store a few thousand patterns without the restriction owing to the data transfer rate, *i.e.* limited only by the readout time.

The improvement in the firmware will also allow the acquisition of pump–probe measurements with up to four separate probe periods, thus reducing the time needed for the experiments and allowing to normalize for systematic fluctuations, *e.g.* in the beam intensity. This is of particular interest for measurements using isolated electron bunches of the synchrotron. In this case the time resolution is given by the electron bunch length and can be down to 20 ps for isolated bunches or even of the order of 100 fs in the case of sliced bunches (Ingold *et al.*, 2007 ▶).

The main disadvantage of the microstrip detector compared with crystal analyzer detectors remains the impossibility of performing an angular and energy selection of the detected X-rays. This prevents the use of MYTHEN for anomalous scattering experiments, increases the background owing to air scattering and reduces the angular (FWHM) resolution owing to the finite size of the samples compared with crystal analyzer detectors.

In the framework of the upgrade of the Materials Science beamline to an undulator source (Willmott *et al.*, 2010 ▶), *i.e.* with higher brightness and smaller focus size, the replacement of the forward detector modules with microstrip detectors with a 25 µm strip pitch is foreseen in order to reduce the contribution arising from the pixel size of the detector in the *d*-spacing resolution (Bergamaschi *et al.*, 2008 ▶).

Feasibility studies aiming at the improvement of the absorption efficiency of the microstrip sensors for X-ray energies larger than 15 keV are being carried out. The tests include high-*Z* material sensors like cadmium telluride (CdTe) (Franchi *et al.*, 2006 ▶) and silicon sensors of thicknesses up to 2 mm.

A front-end ASIC (Mozzanica *et al.*, 2010 ▶) capable of detecting up to 10^4^ X-rays simultaneously with single-photon-counting resolution is under development for X-ray free-electron laser experiments (Blome *et al.*, 2005 ▶). In the meantime, a further upgrade of the front-end single-photon-counting ASIC with a higher readout speed, the possibility of defining an energy window and improved capabilities for pump–probe experiments are being evaluated.    

## Figures and Tables

**Table 1:** Results of the calibration measurements

Settings	Gain (mV keV^−1^)	ENC (e^−^)	2Σ (keV)	τ_d_ (ns)	Φ_max_ (kHz)
Standard	7.11 ± 0.13	230 ± 7	8.3 ± 0.2	170 ± 10	5900 ± 300
		0.83 ± 0.02			
Fast	5.55 ± 0.13	262 ± 7	9.4 ± 0.2	110 ± 10	9000 ± 900
		0.94 ± 0.02			
High gain	9.19 ± 0.22	195 ± 7	7.0 ± 0.2	750 ± 50	1330 ± 90
		0.70 ± 0.02			

**Table 2:** Threshold dispersion over the whole detector for different X-ray energies and detector settings

	Threshold dispersion (eV)					
	Standard		Fast		High gain	
Energy (eV)	Untrimmed	Trimmed	Untrimmed	Trimmed	Untrimmed	Trimmed
8750	1623 ± 6	158 ± 1	1761 ± 7	172 ± 1	1395 ± 6	251 ± 1
12500	1592 ± 6	85.2 ± 0.4	1625 ± 6	106.1 ± 0.4	1393 ± 6	175.9 ± 0.7
17500	1627 ± 6	97.0 ± 0.4	1615 ± 6	128.2 ± 0.5	1449 ± 6	307 ± 1
25000	1656 ± 6	187.0 ± 0.7	1631 ± 7	212.0 ± 0.8	1532 ± 6	582 ± 2

**Table 3:** Wavelength λ, 2θ zero-offset and profile parameters of the structural refinement of Si NIST 640C and WPPM of NAC as returned by *FullProf*

	Si-NIST 640C	NAC	CaF 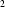 in NAC
λ (Å)	1.000003 (1)	0.495747 (1)	0.495747 (1)
2θ zero-offset	−0.01202 (1)	0.02167 (1)	0.02167 (1)
*U*	0.00029 (1)	0.001487 (1)	0.005221 (1)
*V*	0.00037 (1)	−0.000266 (1)	−0.000841 (1)
*W*	0.00030 (1)	0.000109 (1)	0.000057 (1)
*X*	0.01516 (1)	0.006916 (1)	0.000039 (1)
*Y*	0.00099 (1)	0.000025 (1)	0.000025 (1)
S_L	0.00841 (1)	0.00300 (1)	0.00300 (1)
D_L	0.00427 (1)	0.01190 (1)	0.01190 (1)
